# 基于多壁碳纳米管改进QuEChERS法结合气相色谱-串联质谱法测定云木香中35种禁用农药残留

**DOI:** 10.3724/SP.J.1123.2023.07018

**Published:** 2024-03-08

**Authors:** Congsheng YI, Rui LIU, Zhipeng WU, Bo LIU, Lei DU

**Affiliations:** 中科检测技术服务(广州)股份有限公司, 广东 广州 510650; Chinese Academy of Sciences Testing Technical Services (Guangzhou) Co., Ltd., Guangzhou 510650, China

**Keywords:** 气相色谱-串联质谱, QuEChERS, 多壁碳纳米管, 禁用农药, 云木香, gas chromatography-tandem mass spectrometry (GC-MS/MS), QuEChERS, multi-walled carbon nanotubes, prohibited pesticides, *Saussurea costus*

## Abstract

本研究以中药材云木香中的35种禁用农药为研究对象,利用多壁碳纳米管(MWCNTs)、十八烷基键合硅胶(C_18_)和无水硫酸镁(MgSO_4_)作为净化吸附剂,建立了QuEChERS技术与气相色谱-串联质谱法(GC-MS/MS)相结合的快速检测方法。样品由乙腈提取,经改进的QuEChERS净化后,用GC-MS/MS检测,多反应监测模式(MRM)分析,内标法定量。针对云木香的特点,对比了C_18_、MWCNTs、*N*-丙基乙二胺(PSA)、石墨化炭黑(GCB)4种净化材料不同配比下的净化效果,确定了最优的吸附剂组合为450 mg MgSO_4_、400 mg C_18_和50 mg MWCNTs。该吸附剂组合比传统净化包具有更优异的净化效果,且不会吸附目标物,实验结果稳定可靠。方法学研究表明,35种农药线性良好,相关系数(*r*^2^)均≥0.9970; 3个添加水平下的平均回收率为69.6%~126.9%,相对标准偏差(RSD)均小于10%;检出限(LOD)为0.2~5.4 μg/kg,定量限(LOQ)为0.6~18.1 μg/kg。用该方法检测了20批送检云木香样品中35种农药的残留情况,其中6批样品中检出目标物。本方法简便、灵敏、准确,可用于云木香中农药残留的检测,为云木香的种植生产和质量管控提供技术支撑。

## Abstract (en)

*Saussurea costus*, a perennial herb belonging to the Asteraceae family, is a vital ingredient in traditional Chinese medicine. Increased demands for the herb have led to its widespread cultivation in China, but the corresponding increase in pesticide use has raised concerns about pesticide residues. Such residues would affect the safety and global market potential of *Saussurea costus*. Thus, a simple method is crucial to detect pesticide residues. The QuEChERS technique, in combination with gas chromatography-tandem mass spectrometry (GC-MS/MS), is commonly used for residue detection. However, traditional adsorbents may be unable to purify complex herbal mixtures well, affecting accuracy and instrument performance. Choosing suitable purification materials for *Saussurea costus* samples with complex matrices is of significant importance. This study focused on the detection of 35 prohibited pesticides in *Saussurea costus*. A rapid detection method was established by combining the QuEChERS technique with GC-MS/MS and utilizing a combination of multiwalled carbon nanotubes (MWCNTs), octadecylsilane-bonded silica gel (C_18_), and anhydrous magnesium sulfate (MgSO_4_) as the purification adsorbent. The samples were extracted with acetonitrile, purified by an improved QuEChERS process, subjected to GC-MS/MS analysis in multiple reaction monitoring (MRM) mode, and quantified using the internal standard method. The purification effects of four materials (C_18_, MWCNTs, *N*-propyl ethylenediamine (PSA), and graphitized carbon black (GCB)) and their optimal dosages were investigated by considering the matrix characteristics of the samples. An orthogonal experimental design was employed to optimize the ratio of adsorbent combinations, and the optimal adsorbent combination was determined to be 450 mg of MgSO_4_, 400 mg of C_18_, and 50 mg of MWCNTs. Matrix effect (ME) evaluation of the *S. costus* matrix showed that 31 target compounds strongly exhibited matrix-enhancement effects. Thus, matrix-matched calibration was employed in this study. Methodological investigation revealed that the standard curves for the 35 pesticides exhibited good linearity, with correlation coefficients (*r*^2^) greater than 0.9970. The average recoveries at three spiked levels ranged from 69.6% to 126.9%, and the relative standard deviations (RSDs) for parallel groups were all less than 10%. The limits of detection (LODs) and quantification (LOQs) ranged from 0.2 to 5.4 μg/kg and from 0.6 to 18.1 μg/kg, respectively. The developed method was used to screen and detect 35 pesticide residues in 20 batches of *S. costus* samples, and the target compounds were detected in six batches. The proposed method is simple, sensitive, and accurate. Thus, it is suitable for the rapid screening and detection of the 35 pesticide residues in *S. costus* and provides technical support for the cultivation, production, and quality control of the herb.

云木香(*Saussurea costus*)为菊科木香属多年生高大草本植物,以根入药^[1]^,用于中寒气滞、胸腹胀痛、呕吐、泄泻、里急后重等症^[2]^,其药用历史悠久,是众多中成药(如木香顺气丸、六味木香丸、止痢宁片等)配方的主要原料药材之一^[3]^。该植物原产自印度,自1935年引入云南丽江开始种植,1959年开始出口^[4]^。近年来,随着社会和经济的发展,人们的生活水平逐渐提高,对健康和养生的关注日益增加,对云木香的市场需求也不断上升^[5]^。目前,云木香在云南、广西、四川、甘肃、陕西等省份都有大面积种植,其中仅云南省种植面积就在10万亩左右^[6]^。为了提高产量并减少病虫草害的发生,人们在药材种植过程中大量使用农药,这也导致了药材农药残留风险的增加,给用药的安全性和有效性带来潜在威胁。因此,建立简便、高效的云木香中农药多残留检测方法,以保障云木香原材料的用药安全,变得非常重要。

QuEChERS前处理方法具有快速、高通量、高效和低成本等优点,与气相色谱-串联质谱(GC-MS/MS)检测技术相结合,可以满足高通量、高选择性和高灵敏度的检测技术要求^[7⇓-9]^,已被广泛应用于农药监督机构的各种评估和验证试验的前处理中^[10]^。然而,在处理复杂中药基质时,常用的吸附剂,如PSA、C_18_和GCB等,并未展现出理想的净化效果。部分目标物可能会与杂质一起被吸附,这不仅影响样品检测结果的准确性,还可能导致仪器污染,降低仪器的检测性能^[11]^。因此,需要针对复杂中药基质寻找更适合的吸附剂,以提高净化效果并确保分析的准确性和仪器的性能。因此,为了保证复杂样品农药残留的精准分析,根据基质特点选择适用的净化吸附剂显得尤为关键。

近年来,研究人员根据复杂基质中杂质成分的特点,相继使用了多种新型净化材料,并取得了不错的效果。例如,磁性氧化锆(Fe_3_O_4_-ZrO_2_)可有效去除人参中氨基酸、脂肪酸、人参皂苷等杂质^[12]^,六方氮化硼(h-BN)纳米材料能有效去除草莓中的脂肪酸、色素等杂质^[13]^,可提高农残检测的灵敏度和可靠性。多壁碳纳米管(MWCNTs)作为吸附材料,其管体由石墨组成,具备较好的疏水性能,并且其具有的多孔结构进一步增大了比表面积,从而赋予其强大的吸附能力,可以去除植物源性样品中色素、甾醇、糖类、有机酸和酚类等杂质,逐渐在农药残留检测领域引起关注^[14⇓-16]^。针对含有大量生物碱、甾醇、酚类和挥发油的云木香^[17]^,本研究尝试使用MWCNTs、C_18_和无水硫酸镁(MgSO_4_)作为组合吸附剂,探索其对云木香基质的净化效果。

目前,关于云木香中农残检测的文献报道较少。本文选取国家禁用的35种农药作为检测目标物,以《中国药典》2020年版四部通则为指导^[18]^,将MWCNTs、C_18_和MgSO_4_作为吸附剂组合,利用改进的QuEChERS前处理方法,结合GC- MS/MS,建立了同时检测云木香中35种禁用农药残留的方法。

## 1 实验部分

### 1.1 仪器、试剂与材料

TQ8040气相色谱-三重四极杆质谱联用仪(配有电子轰击源(EI))、ATX124型电子分析天平(日本岛津公司); Milli-Q超纯水器(美国Millipore公司); BC-1000型多管旋涡混合仪(深圳逗点公司); TGL-16M型高速冷冻离心机(上海卢湘仪公司); MTN-5800型氮吹浓缩装置(天津奥特赛恩公司)。

提取盐包(内含6 g MgSO_4_和1.5 g无水乙酸钠(NaAc))、C_18_、GCB、PSA(江苏科普诺化工科技有限公司), MWCNTs(长度10~30 μm,外径20~30 nm,纯度>98%,比表面积>110 m^2^/g,天津博纳艾杰尔科技有限公司),中药净化包(900 mg MgSO_4_、300 mg PSA、300 mg C_18_、300 mg硅胶、90 mg GCB,市售)。乙腈(色谱纯,上海安谱实验科技股份有限公司),乙酸、无水硫酸镁(分析纯,上海阿拉丁生化科技股份有限公司)。

35种农药混合标准溶液、磷酸三苯酯(内标)标准溶液(各浓度见表1)均购于坛墨质检科技股份有限公司。

**表 1:** 35种农药及内标的保留时间、质谱参数及其质量浓度

No.	Pesticide	Retention time/min	Quantitative ion pair (m/z)	Qualitative ion pairs (m/z)	CEs/eV	C/ (μg/mL)
1	demeton-O (内吸磷-O)	11.92	88.0>60.0	88.0>59.0, 88.0>45.0	4, 20, 25	20
2	ethoprophos (灭线磷)	12.40	199.7>157.8	157.8>96.7, 157.8>113.8	5, 20, 15	40
3	chlordimeform (杀虫脒)	12.75	152.0>117.0	196.0>181.0	15, 5	40
4	sulfotep (治螟磷)	12.95	322.0>174.0	322.0>294.0, 322.0>202.0	15, 10, 20	40
5	phorate (甲拌磷)	13.12	260.0>75.0	230.8>175.0, 230.8>128.6	5, 10, 25	40
6	α-hexachlorocyclohexane (α-六六六)	13.47	181.0>145.0	218.7>182.9, 218.9>147.0	15, 5, 10	100
7	terbufos (特丁硫磷)	13.73	230.8>129.0	230.8>175.0, 230.8>203.0	25, 13, 5	40
8	demeton-S (内吸磷-S)	13.79	88.0>60.0	88.0>59.0, 88.0>45.0	4, 20, 25	20
9	γ-hexachlorocyclohexane (γ-六六六)	14.44	181.0>145.0	218.7>182.9, 218.9>147.0	15, 5, 10	100
10	monocrotophos (久效磷)	14.48	127.0>109.0	127.0>95.0, 127.0>79.0	12, 16, 20	60
11	fipronil-desulfinyl (氟甲腈)	14.77	388.0>333.0	388.0>281.0	20, 35	40
12	β-hexachlorocyclohexane (β-六六六)	15.06	181.0>145.0	218.7>182.9, 218.9>147.0	15, 5, 10	100
13	δ-hexachlorocyclohexane (δ-六六六)	15.71	181.0>145.0	218.7>182.9, 218.9>147.0	15, 5, 10	100
14	aldrin (艾氏剂)	15.81	263.0>193.0	276.8>240.7, 276.8>169.7	35, 10, 35	100
15	parathion-methyl (甲基对硫磷)	16.11	263.1>109.0	263.1>136.0, 263.1>79.0	13, 5, 35	40
16	fipronil-sulfoxide (氟虫腈亚砜)	16.43	420.0>351.0	420.0>255.0	12, 20	40
17	dicofol (三氯杀螨醇)	16.44	250.0>139.0	139.0>111.0, 250.0>215.0	15, 15, 5	100
18	fipronil (氟虫腈)	16.52	367.0>213.0	351.0>255.0, 367.0>255.0	35, 20, 25	40
19	parathion (对硫磷)	16.70	291.0>109.0	291.0>81.0, 139.0>109.0	25, 30, 10	100
20	2,4'-dicofol (2,4'-三氯杀螨醇)	16.98	250.0>139.0	139.0>111.0, 250.0>215.0	15, 15, 5	100
21	isofenphos-methyl (甲基异柳磷)	17.03	241.0>120.8	241.0>199.0, 241.0>166.7	20, 5, 10	40
22	isocarbophos (水胺硫磷)	17.42	135.7>108.0	120.7>65.0, 121.0>93.0	15, 20, 15	100
23	fipronil-sulfone (氟虫腈砜)	17.92	383.0>255.0	383.0>213.0, 452.0>383.0	20, 32, 8	40
24	α-endosulfan (α-硫丹)	17.93	240.8>205.6	240.8>170.0, 194.8>159.0	15, 25, 10	100
25	p,p'-dichlorodiphenyldichloroethylene (4,4'-滴滴伊)	18.37	246.0>176.0	316.0>246.0, 246.0>210.0	30, 25, 28	100
26	fenamiphos (苯线磷)	18.58	303.1>122.0	303.1>154.0, 303.1>195.0	20, 30, 25	40
27	dieldrin (狄氏剂)	18.59	263.0>193.0	276.8>240.7, 276.8>169.7	35, 10, 35	100
28	Posfolan-methyl (甲基硫环磷)	19.06	168.0>109.0	227.0>92.0	15, 10	60
29	nitrofen (除草醚)	19.44	201.8>138.7	282.8>253.0, 282.8>201.8	28, 10, 15	100
30	o,p'-dichlorodiphenyltrichloroethane (2,4'-滴滴涕)	19.45	235.0>165.0	235.0>199.0, 237.0>165.0	25, 15, 25	100
31	p,p'-dichlorodiphenyldichloroethane (4,4'-滴滴滴)	19.62	235.0>165.0	237.0>165.0, 235.0>199.0	25, 25, 18	100
32	β-endosulfan (β-硫丹)	19.88	206.8>171.8	194.8>124.7, 194.8>159.0	15, 30, 10	100
33	p,p'-dichlorodiphenyltrichloroethane (4,4'-滴滴涕)	20.18	235.0>165.0	235.0>199.0, 237.0>165.0	25, 18, 25	100
34	endosulfan sulfate (硫丹硫酸酯)	20.80	271.8>236.7	273.8>238.9, 271.8>141.0	15, 15, 40	100
35	coumaphos (蝇毒磷)	23.89	361.8>109.0	361.8>81.0, 361.8>225.8	16, 32, 14	100
36	triphenyl phosphate (磷酸三苯酯, IS)	21.27	326.0>233.0	326.0>215.0, 326.0>169.0	10, 25, 30	100

CE: collision energy.

云木香样品:客户送检。

### 1.2 实验条件

#### 1.2.1 色谱条件

色谱柱:SH-Rxi-17Sil MS(30 m×0.25 mm×0.25 μm);升温程序:初始温度80 ℃,保持1 min,以10 ℃/min升温至310 ℃,保持5 min;进样口温度为220 ℃;载气为氦气,纯度≥99.999%,流速2.20 mL/min,恒压压力146.0 kPa;进样方式为不分流进样,进样量为1 μL。

#### 1.2.2 质谱条件

离子源:电子轰击源;一级质谱电离能:70 eV;接口温度250 ℃;离子源温度230 ℃;检测器电压0.4 kV;溶剂延迟时间3.2 min;多反应监测(MRM)模式。35种农药及内标的质谱参数见表1。

### 1.3 样品前处理

称取过筛(内径(355±13) μm)后的样品粉末3 g(精确至0.01 g),置于50 mL聚苯乙烯具塞离心管中,加入1%乙酸溶液15 mL,涡旋使药粉充分浸润,放置30 min,然后加入乙腈15 mL,涡旋混匀,涡旋振荡5 min(2000 r/min),接着加入提取盐包,立刻摇散,并在冰浴中冷却10 min,再进行涡旋振荡5 min(2500 r/min)。随后进行离心5 min(9000 r/min),取上清液9 mL,置于离心管(含450 mg MgSO_4_、400 mg C_18_、50 mg MWCNTs)中,再次进行涡旋5 min(1800 r/min),然后离心5 min(9000 r/min)。精密吸取上清液5 mL,置于氮吹仪上,在40 ℃水浴中浓缩至约0.4 mL,加入100 μL磷酸三苯酯使用液,用乙腈稀释至总体积1.0 mL,过膜备用。

### 1.4 标准溶液的配制

取35种混合标准溶液0.5 mL,置于10 mL容量瓶中,用乙腈溶解并稀释至刻度,摇匀,制成混合标准储备液,-20 ℃保存。

取100 μL磷酸三苯酯标准溶液置于10 mL容量瓶中,用乙腈溶解并稀释至刻度,摇匀,制成质量浓度为1 μg/mL的磷酸三苯酯内标使用液,-20 ℃保存。

取云木香空白样品,按照1.3节方法处理,并分成5份,在浓缩液中各加入100 μL内标使用液,随即分别加入35种农药混合标准储备液0.01、0.02、0.05、0.10、0.20 mL,用乙腈定容至1.0 mL,混匀,制成基质混合标准工作溶液。

## 2 结果与讨论

### 2.1 质谱条件的选择

根据GC-MS/MS的多反应监测模式,本研究通过设定多个时间段和扫描通道同时分析35种农药及内标。首先,对目标物的混合标准溶液在*m/z* 45~500范围内进行全扫描,并利用NIST标准库匹配检索,以确定目标物的出峰时间和一级碎片离子。为了选择最适合的母离子,选取离子强度高的一级碎片离子作为母离子,然后通过优化碰撞能量获得子离子。最后采用MRM模式对目标物进行检测和分析。在此条件下,35种农药及内标的总离子流图(TIC)见图1。

**图 1:**
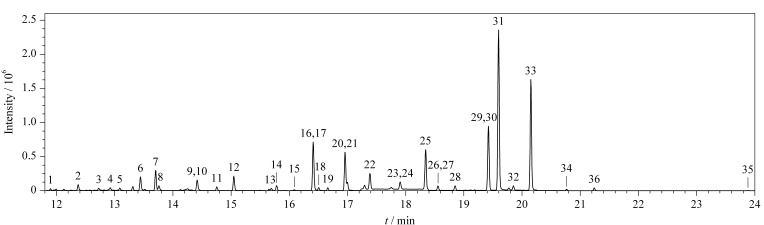
35种农药及内标的总离子流色谱图

### 2.2 浸泡时间、提取时间、净化时间的优化

在进行云木香样品的提取前,需在样品中加入1%乙酸溶液充分浸泡,以使样品膨胀松散,确保提取溶剂能够与样品基质充分接触,并减弱目标物与样品基质之间的相互作用^[19]^。为研究不同浸泡时间对回收率的影响,本实验对加标后的空白样品分别浸泡了10、20、30、40、50 min。结果显示,在不同浸泡时间下,目标物的平均回收率分别为85.9%、91.6%、95.2%、94.8%、94.3%。随着浸泡时间的延长,提取液的颜色逐渐加深。研究表明,浸泡时间越长,共萃物的提取量越多。在浸泡时间为10~30 min时,目标物的平均回收率随着浸泡时间的增加而显著增加;而在浸泡时间为30~50 min时,回收率稍有下降。此外,本研究还探究了不同的提取时间和净化时间(均为1、3、5、7、9 min)对目标物平均回收率的影响。在提取时间和净化时间为1~5 min时,平均回收率显著增加,超过5 min后回收率趋于稳定,不会明显变化。综合考虑了35种农药的回收率、实验效率和能耗节约等因素,最终选择浸泡时间为30 min,提取时间和净化时间均为5 min。

### 2.3 净化材料的优化

本研究基于前期的大量实验,固定无水MgSO_4_为450 mg,并考察了18组吸附剂(见表2)对云木香样品的净化效果。分别考察了不同C_18_添加量的净化效果(Y1~Y3组)。结果显示,随着C_18_添加量增加,残余的油性物质逐渐减少,如图2a所示。随后,考察了C_18_的添加量(Y4~Y8组)对农药回收率的影响(图2b)。当C_18_用量增至400 mg时,农药的平均回收率达到最高(95.8%),但随着用量再次增加,农药平均回收率逐渐下降。实验结果表明,C_18_中的长烷基链能够有效吸附脂溶性成分和大分子物质(例如挥发油、多糖、菇类、蛋白质等),但使用过量的C_18_也会吸附脂溶性农药。

**表 2:** 不同吸附剂的组成

No.	MgSO_4_/mg	C_18_/mg	MWCNTs/mg	GCB/mg	PSA/mg
Y1	450	0	0	0	0
Y2	450	200	0	0	0
Y3	450	400	0	0	0
Y4	450	0	50	0	0
Y5	450	200	50	0	0
Y6	450	400	50	0	0
Y7	450	500	50	0	0
Y8	450	600	50	0	0
Y9	450	400	0	0	0
Y10	450	400	10	0	0
Y11	450	400	30	0	0
Y12	450	400	70	0	0
Y13	450	400	90	0	0
Y14	450	400	0	0	150
Y15	450	400	50	10	0
Y16	450	400	50	20	0
Y17	450	400	50	30	0
Y18	450	400	50	40	0

**图 2:**
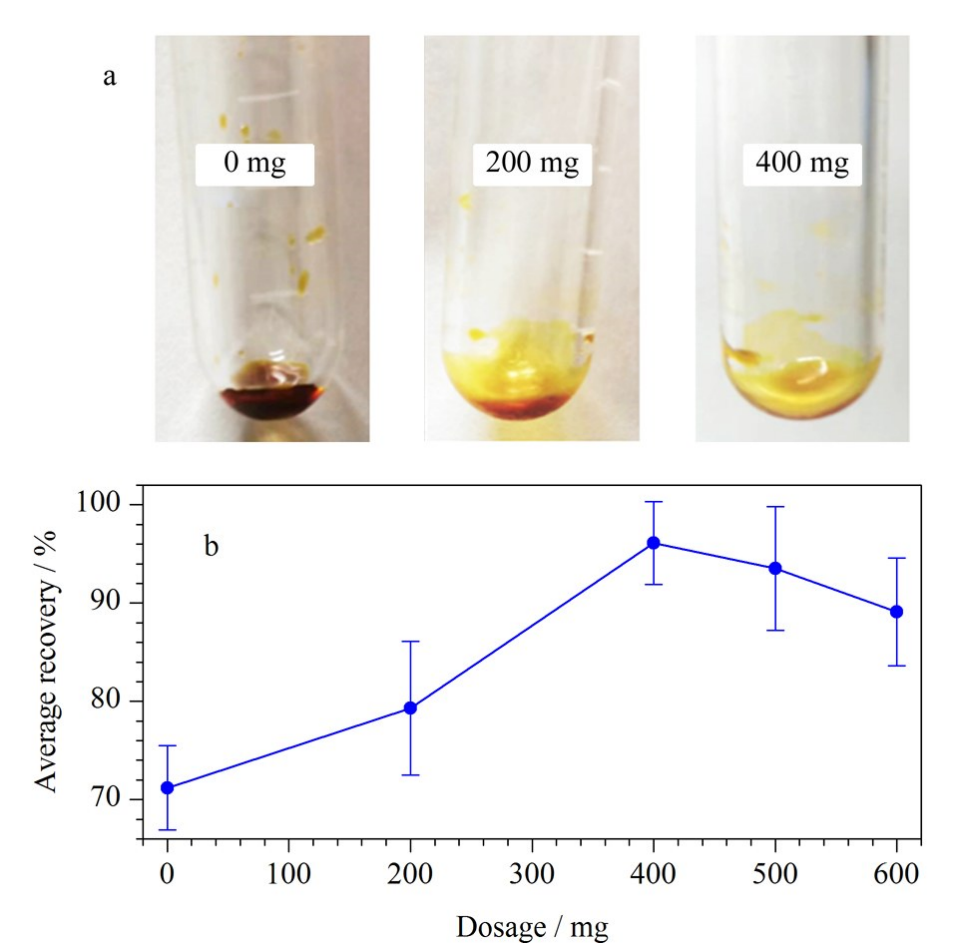
C_18_的添加量对(a)油性物残留量和(b)35种农药回收率的影响(*n*=3)

为了评估不同MWCNTs用量对云木香提取液的净化效果及回收率的影响,本实验采用Y6、Y9~Y13组吸附剂组合分别对空白加标样品净化处理。观察待测液(如图3a)的外观变化,随着MWCNTs添加量的增加,净化液颜色逐渐变浅,增至50 mg后未见明显变化,这表明MWCNTs对于净化云木香样品中的杂质具有良好的效果。如图3b所示,当MWCNTs用量增至50 mg时,平均回收率达到95.8%,但进一步增加MWCNTs用量后35种农药的平均回收率未见明显变化。综合考虑回收率和云木香样品的净化效果,最终确定将MWCNTs用量设定为50 mg。

**图 3:**
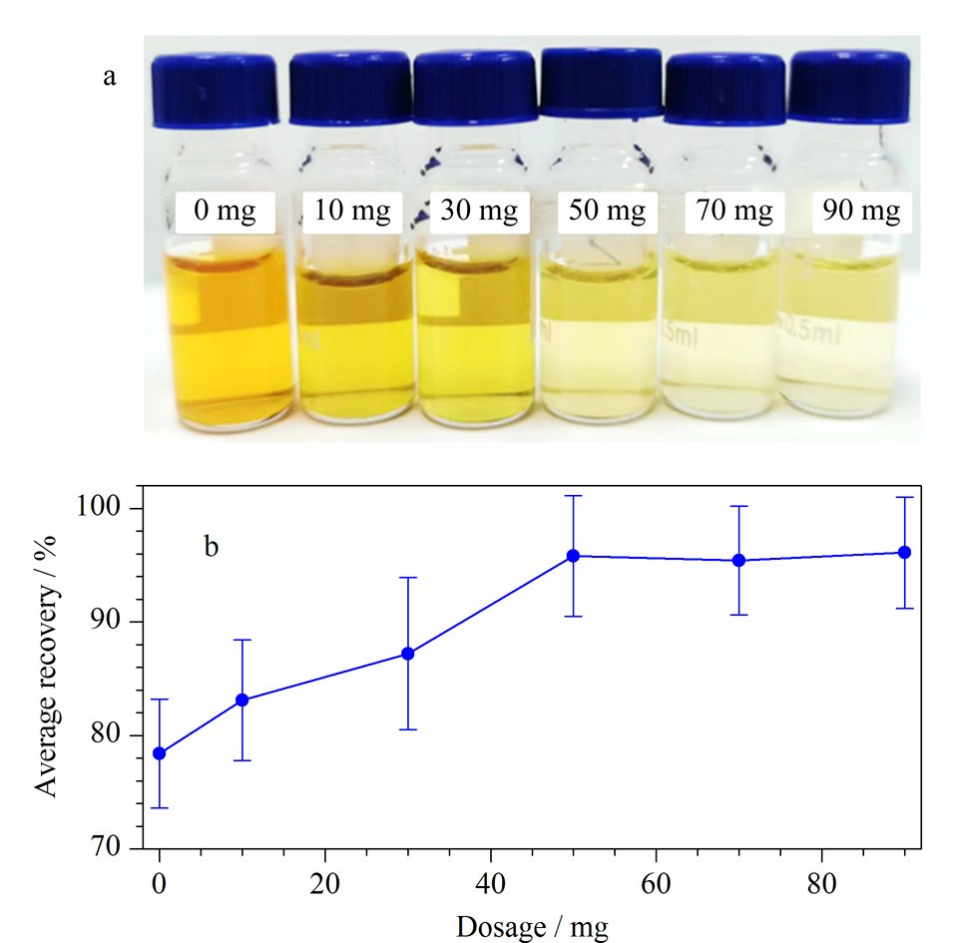
MWCNTs的添加量对(a)净化后颜色和(b)35种农药回收率的影响(*n*=3)

此外,已有研究证明PSA与MWCNTs都可以去除酚类、有机酸、色素及糖类等杂质^[20,21]^。本研究进一步比较了MWCNTs与PSA在云木香样品提取液净化效果上的差异。实验选用Y11与Y14两组吸附剂组合对比处理,其中Y11组中MWCNTs用量为Y14组中PSA用量的1/5。结果表明,MWCNTs净化的农药化合物回收率整体更高,且回收率为70%~120%的农药数量更多,MWCNTs吸附色素的能力也优于PSA。这证明MWCNTs比PSA具有更好的净化效果,而且价格更低廉且用量更少。

为了进一步优化吸附剂的组成,本研究在吸附剂中加入不同量的GCB(Y6、Y15~Y18组)。实验结果显示,随着GCB添加量的增加,杀虫脒、蝇毒磷、特丁硫磷的回收率明显降低,而其他目标物的回收率未见明显变化。这表明GCB对这3种农药有较强的吸附作用,并且其主要作用是吸附基质中的色素。然而,经过MWCNTs与C_18_净化后的待测液中色素含量已经较少,因此在组合吸附剂中无需再添加GCB。

综上所述,本研究选择Y6组(450 mg MgSO_4_、 400 mg C_18_、50 mg MWCNTs)作为云木香的基质净化吸附剂组合。经过这种吸附剂组合的净化,可以高效去除云木香样品中的各类杂质和色素,同时避免过多吸附目标化合物。

### 2.4 基质效应

基质效应(ME)指的是相同浓度的目标分析物(标准品)在纯溶剂和在空白基质中的响应差异。基质效应一般会导致化合物响应信号的增强或抑制^[22]^。本文中采用较为常用的相对响应值法来评价ME,即ME=*B/A*×100%,其中*A*为纯溶剂中农药的响应值,*B*为样品基质中添加相同浓度农药的响应值。当ME<80%时视为强基质抑制效应,当80%≤ME≤120%时视为弱基质效应,当ME>120%时视为强基质增强效应^[23]^。

实验结果表明,35种农药中31种表现为强基质增强效应,为弥补基质效应产生的差异,本研究采用基质匹配标准曲线法降低基质效应的影响。

### 2.5 方法学评价

#### 2.5.1 标准曲线和定量限

对基质混合标准工作溶液进行测定,以各农药的含量为横坐标(*x*, μg/kg),目标物与内标峰面积之比为纵坐标(*y*),绘制标准曲线。结果表明, 35种农药的线性关系良好,相关系数(*r*^2^)均≥0.9970(表3)。以空白样品加标处理并用本方法进行检测,LOD(*S/N*=3)为0.2~5.4 μg/kg, LOQ(*S/N*=10)为0.6~18.1 μg/kg。满足《中国药典》2020年版药材及饮片(植物类)禁用农药定量限的要求^[18]^。

**表 3:** 35种农药的线性范围、相关系数、检出限、定量限、加标回收率和相对标准偏差(*n*=6)

Pesticide	Linear range/ (μg/kg)	r^2^	LOD/ (μg/kg)	LOQ/ (μg/kg)	Recoveries (RSDs)/%		
					Low	Medium	High
Demeton-O	10-200	0.9996	2.4	8.1	101.5 (6.7)	89.6 (4.2)	88.9 (4.5)
Ethoprophos	20-400	0.9991	1.5	5.1	102.0 (5.4)	86.7 (3.8)	86.9 (4.7)
Chlordimeform	20-400	0.9994	2.7	9.1	78.8 (1.9)	75.3 (5.3)	82.6 (1.8)
Sulfotep	20-400	0.9993	2.3	7.5	114.4 (3.5)	107.0 (2.8)	105.7 (4.7)
Phorate	20-400	0.9997	3.1	10.3	107.0 (7.8)	94.4 (4.4)	93.5 (4.7)
α-Hexachlorocyclohexane	50-1000	0.9993	2.0	6.6	123.1 (4.7)	125.7 (2.6)	111.1 (3.6)
Terbufos	20-400	0.9993	3.4	11.2	91.5 (4.7)	85.1 (4.1)	87.3 (5.2)
Demeton-S	10-200	0.9992	2.2	7.3	95.2 (3.6)	84.0 (3.4)	88.0 (6.0)
γ-Hexachlorocyclohexane	50-1000	0.9993	0.6	1.9	119.1 (2.3)	115.6 (6.4)	116.0 (2.4)
Monocrotophos	30-600	0.9990	3.6	12.0	71.3 (6.0)	97.1 (7.5)	120.7 (2.6)
Fipronil-desulfinyl	20-400	0.9998	0.2	0.6	116.9 (3.8)	89.6 (5.9)	96.8 (1.4)
β-Hexachlorocyclohexane	50-1000	0.9998	2.7	8.9	124.8 (4.8)	116.4 (2.2)	111.3 (3.1)
δ-Hexachlorocyclohexane	50-1000	0.9997	1.1	3.8	105.4 (6.3)	100.1 (3.0)	116.3 (2.5)
Aldrin	50-1000	0.9997	1.7	5.8	93.7 (5.1)	85.3 (3.0)	84.7 (5.6)
Parathion-methyl	20-400	0.9994	2.5	8.3	87.5 (3.9)	84.4 (3.0)	85.5 (3.3)
Fipronil-sulfoxide	20-400	0.9990	0.2	0.7	115.5 (8.2)	115.2 (4.7)	117.1 (6.9)
Dicofol	50-1000	0.9997	0.4	1.4	118.5 (9.2)	105.2 (2.5)	109.4 (2.1)
Fipronil	20-400	0.9996	2.8	9.4	101.6 (7.0)	97.5 (3.7)	99.4 (6.2)
Parathion	50-1000	0.9991	3.3	11.0	126.9 (2.9)	122.1 (1.4)	116.4 (3.2)
2,4'-Dicofol	50-1000	0.9994	0.6	2.1	119.1 (3.6)	122.0 (2.2)	104.8 (1.6)
Isofenphos-methyl	20-400	0.9992	0.3	1.1	112.0 (6.0)	114.3 (4.6)	111.0 (4.9)
Isocarbophos	50-1000	0.9970	4.0	13.2	78.1 (6.7)	95.5 (2.4)	91.4 (1.7)
Fipronil-sulfone	50-1000	0.9996	0.8	2.8	106.2 (6.4)	93.3 (4.6)	92.3 (2.9)
α-Endosulfan	20-400	0.9999	0.9	3.1	81.9 (2.7)	85.0 (3.4)	81.0 (3.7)
p,p'-Dichlorodiphenyldichloroethylene	50-1000	0.9999	0.5	1.8	84.2 (6.2)	76.0 (5.0)	73.5 (4.9)
Fenamiphos	20-400	0.9992	1.6	5.2	88.0 (3.5)	89.1 (5.3)	81.8 (1.8)
Dieldrin	50-1000	0.9999	2.3	7.8	102.5 (5.2)	96.8 (3.3)	93.1 (8.4)
Posfolan-methyl	30-600	0.9991	4.4	14.7	88.1 (5.7)	99.4 (3.3)	100.2 (5.7)
Nitrofen	50-1000	0.9995	1.2	4.1	69.8 (7.2)	70.0 (1.6)	69.6 (3.2)
o,p'-Dichlorodiphenyltrichloroethane	50-1000	0.9999	2.3	7.6	95.0 (2.7)	89.0 (4.4)	85.8 (2.7)
p,p'-Dichlorodiphenyldichloroethane	50-1000	0.9993	2.1	6.9	123.8 (3.6)	118.4 (1.7)	115.3 (2.2)
β-Endosulfan	50-1000	0.9998	3.3	10.9	98.0 (5.5)	95.5 (2.9)	91.8 (2.0)
p,p'-Dichlorodiphenyltrichloroethane	50-1000	0.9998	0.4	1.2	115.7 (4.7)	110.8 (3.3)	108.1 (1.1)
Endosulfan sulfate	50-1000	0.9997	3.8	12.7	99.0 (7.3)	104.4 (5.3)	86.3 (3.8)
Coumaphos	50-1000	0.9997	5.4	18.1	96.9 (6.0)	93.0 (9.9)	94.5 (2.5)

#### 2.5.2 准确度和精密度

在空白样品中分别添加混合标准储备液60、150和300 μL,每个水平重复6次,按该研究建立的方法进行测定。添加回收试验结果表明(表3),在3个加标水平下,35种农药的平均回收率为69.6%~126.9%,相对标准偏差均小于10%(*n*=6)。

### 2.6 与传统QuEChERS方法的比较

为了比较本研究设计的改进吸附剂组合与传统吸附剂组合对云木香样品的净化效果,本实验选用了某知名公司的市售传统中药净化包。根据该商业净化包的使用说明,采用《中国药典》2020年(通则2341农药残留量测定法)中第五法 药材及饮片(植物类)禁用农药多残留测定法^[18]^。该方法与本研究中1.3节的前处理方法的提取步骤和净化步骤完全一致,并且处理所需的时间也相同。对云木香样品分别采用这两种方法处理后,使用GC-MS/MS进行测试。

如图4a所示,未净化的云木香原样总离子流色谱图中主要有A、B、C 3个杂质峰。经传统方法净化后(图4b), 3个杂质峰的响应值均得到不同程度的减弱,而经改进方法净化后的B、C峰减弱程度更为明显,且A峰基本消失(图4c)。可见,改进的组合吸附剂对云木香样品的杂质有更好的吸附效果。

**图 4:**
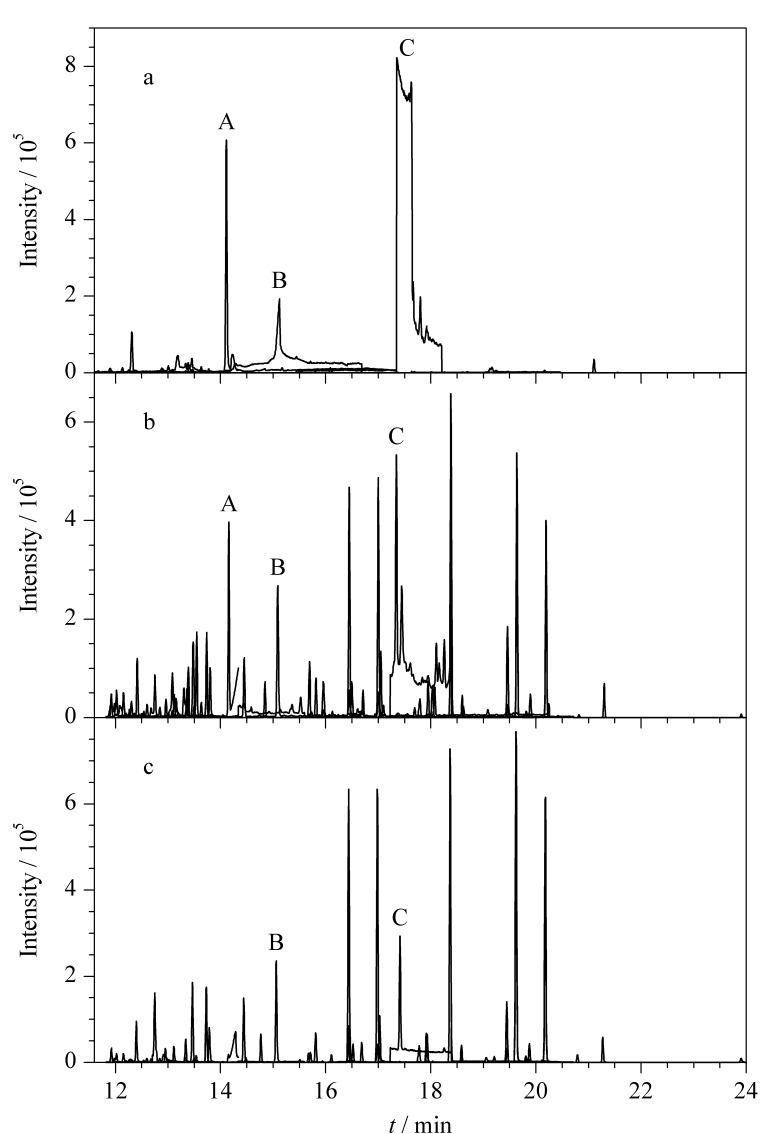
(a)空白样品净化前、(b)加标样品传统方法净化后 和(c)加标样品改进方法净化后35种农药的总离子流色谱图

本实验还比较了改进方法与传统方法的加标回收率和定量限。结果显示,改进方法的回收率总体上高于传统方法,尤其是杀虫脒、蝇毒磷、特丁硫磷这3种农药。由于市售中药净化包中含有90 mg的GCB,正如前文所述,GCB对这3种农药具有较强的吸附作用,因此市售中药净化包并不适用于云木香中杀虫脒、蝇毒磷、特丁硫磷的测定。除杀虫脒、蝇毒磷、特丁硫磷外,传统方法对其余32种农药目标物的LOQ为2.1~41.5 μg/kg;而改进方法对35种农药目标物检测的LOQ为0.6~18.1 μg/kg。显然,改进方法在灵敏度方面表现得更为优越。

结果表明,改进组合吸附剂对云木香的净化表现更为优异,且价格远低于市售中药净化包,因此在云木香农残检测的推广应用上具有显著优势。

### 2.7 实际样品的检测

利用建立的检测方法对送检的20批云木香样品进行测定,结果表明,在6批样品中共检出6种禁用农药。其中,灭线磷、杀虫脒、*α*-六六六的检出率均为10%, *α*-硫丹、狄氏剂、甲基硫环磷的检出率均为5%。各农药的检出含量如下:灭线磷0.16~0.21 mg/kg、杀虫脒0.09~0.11 mg/kg、*α*-六六六0.12~0.37 mg/kg、*α*-硫丹0.13 mg/kg、狄氏剂0.37 mg/kg、甲基硫环磷0.22 mg/kg。图5为6批次阳性样品中部分检出农药的MRM色谱图。

**图 5:**
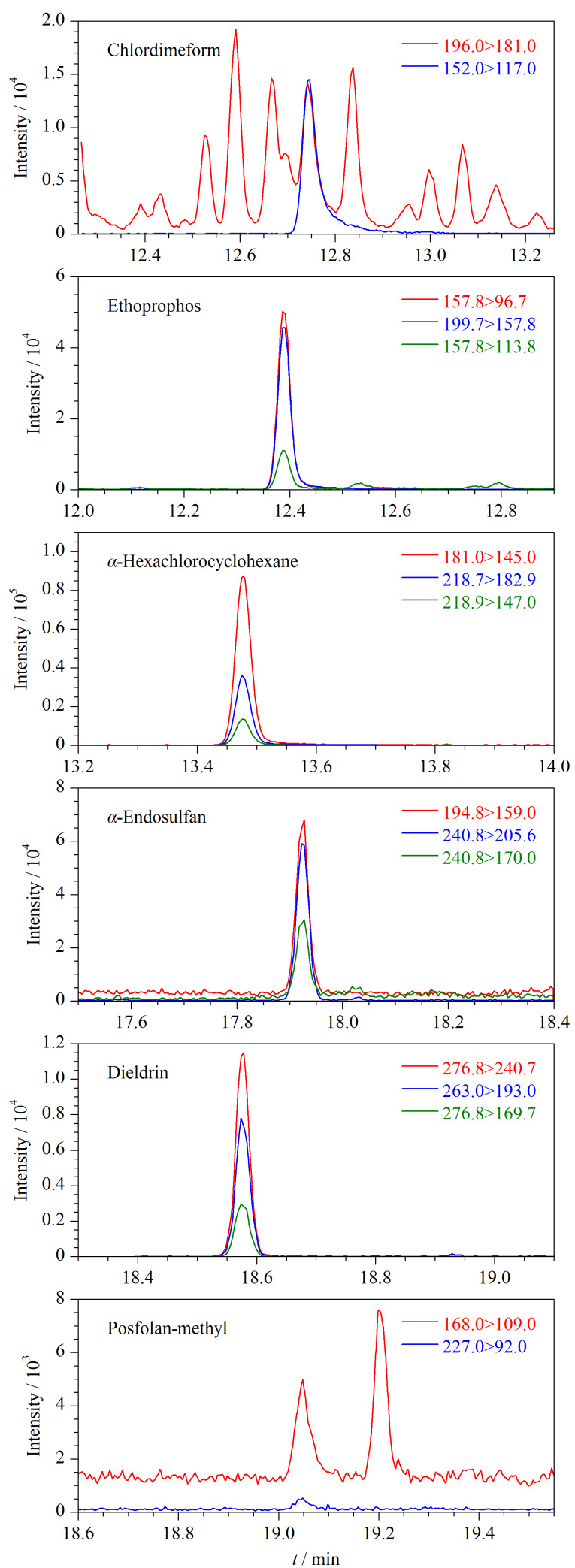
6批次阳性样品中部分检出农药的MRM色谱图

## 3 结论

本研究采用改进的QuEChERS前处理方法,结合GC-MS/MS建立了同时检测云木香中35种禁用农药残留的方法。采用450 mg MgSO_4_、400 mg C_18_、50 mg MWCNTs作为吸附剂组合,有效解决了云木香样品前处理过程中部分化合物回收率低及净化效果差的问题,并利用GC-MS/MS对35种目标物进行高通量检测。本方法具有简便、灵敏、准确以及成本低等优点,可为云木香的种植生产和质量管控提供可靠的技术支撑。
